# Protection Efficacy of the Extract of* Ginkgo biloba* against the Learning and Memory Damage of Rats under Repeated High Sustained +Gz Exposure

**DOI:** 10.1155/2016/6320586

**Published:** 2016-03-16

**Authors:** Liang-En Chen, Feng Wu, Andong Zhao, Hua Ge, Hao Zhan

**Affiliations:** Institute of Aviation Medicine, Air Force, Beijing 100142, China

## Abstract

Repeated high sustained positive Gz (+Gz) exposures are known for the harmful pathophysiological impact on the brain of rats, which is reflected as the interruption of normal performance of learning and memory. Interestingly, extract of* Ginkgo biloba* (EGb) has been reported to have neuroprotective effects and cognition-enhancing effects. In this study, we are interested in evaluating the protective effects of EGb toward the learning and memory abilities. Morris Water Maze Test (MWM) was used to evaluate the cognitive function, and the physiological status of the key components in central cholinergic system was also investigated. Our animal behavioral tests indicated that EGb can release the learning and memory impairment caused by repeated high sustained +Gz. Administration of EGb to rats can diminish some of the harmful physiological effects caused by repeated +Gz exposures. Moreover, EGb administration can increase the biological activities of superoxide dismutase (SOD) and glutathione peroxidase (GSH-Px) but reduce the production of malondialdehyde (MDA). Taken together, our study showed that EGb can ameliorate the impairment of learning and memory abilities of rats induced by repeated high sustained +Gz exposure; the underlying mechanisms appeared to be related to the signal regulation on the cholinergic system and antioxidant enzymes system.

## 1. Introduction

Positive Gz (+Gz) is defined as the acceleration along the foot-to-head direction, which will cause the blood to flow toward the direction of inertia force, make the blood be redistributed to the lower body, and eventually lead to cerebral ischemia. Thus, high sustained +Gz seriously threatens the health of pilots. Particularly, in some extreme cases, pilots of modern high performance fighters have to suffer the produced acceleration as high as +9 Gz for 15 to 45 s, and this can cause loss of consciousness (G-LOC) of the pilots [1]. In animal experiments, this circumstance also results in G-LOC for rats and mice [2]. Moreover, recent studies showed that cerebral ischemia induced by high sustained +Gz may damage the ability of learning and memory in animals [3, 4]. Therefore, it is important to explore ameliorating brain function drugs to counteract the harm of high sustained +Gz.

Extracts of* Ginkgo biloba* (EGb) leaf have been used for centuries in traditional Chinese medicine, which can prevent transient global ischemia-induced delayed neuronal death and improve the learning and memory deficit. EGb is effective to improve both short-term [5, 6] and long-term memory in both young and aged healthy humans [7, 8]. EGb exhibited positive efficacy in treating impairments and age-associated dementia, cerebral insufficiency [9]. Consistent with the clinical efficacy, EGb showed efficacy in established animal models on the release of the learning and memory impairment. There are clear lines of evidence showing that EGb can improve the learning and memory deficit induced by both aging [10] and various stimuli, such as chronic stress [11, 12], ischemia [13], amyloid [14], scopolamine [15], and aluminum [16] in male rats.

It was reported that EGb improves cognitive function through the interaction with the antioxidant and cholinergic systems [17]. In this study, we investigated the efficacy of standardized* Ginkgo biloba* extracts (EGb761) on the +Gz-induced cognitive impairment and antioxidant and cholinergic systems changes in the hippocampus of the tested animals.

## 2. Materials and Methods

### 2.1. Chemicals

EGb761 was manufactured by Dr. Willmar Schwabe GmbH & Co. (Karlsruhe, Germany). It contains 24%* Ginkgo* flavonole glycosides and 6% terpenoids. EGb761 was dissolved in saline and administered orally using gavage. The doses were adjusted such that each animal received a volume of 20 mL·kg^−1^. ACh, ChAT, AChE, SOD, GSH-Px, and MDA assay kits were purchased from Nanjing Jiancheng Bioengineering Institute (Nanjing, China). All other reagents used in this study were purchased from Boster Biological Technology, Ltd. (Wuhan, China), unless otherwise indicated.

### 2.2. Animals and Drug Treatment

All experiments and procedures performed in this study were reviewed and approved by the Animal Care and Use Committee of the Institute of Aviation Medicine, Air Force (IACUC approval number 20141010). Sixty male Sprague-Dawley rats, 8 weeks old, 280 ± 20 g in weight, were obtained from Vital River Laboratory Animal Technology Co., Ltd. (Beijing, China). Rats were randomly assigned to five groups, control group, model group, and three EGb761-treated groups (EGb-L, EGb-M, and EGb-H, at 50, 100, and 200 mg/kg, resp.), with 12 rats in each. All drugs were orally administered to rats daily for 14 days with the doses listed above. The model and the control group were treated with the vehicle. Body weights of rats were recorded weekly.

### 2.3. Animals Acceleration Exposure

From the 8th to 14th days after the start of oral administration, animals were exposed to acceleration. The animal centrifuge was 2 m in radius and was capable of producing a gravity range from +1 Gz to +15 Gz, with an onset rate of 0.1–6 Gz/s. Each rat was put into a 15 cm × 5 cm × 3 cm Plexiglass box which was clamped to the centrifuge arm with its head facing toward the axis of the centrifuge for +Gz orientation. Rats in model and EGb761-treated groups were exposed to +10 Gz for 5 min as reported elsewhere [18]. The onset/offset rate was +1 G/s. Meanwhile, nothing was done to the control group.

### 2.4. Morris Water Maze Test (MWM)

This task was adapted for rats from the prototype originally described by Morris [19] with some modifications. The MWM was performed on the eighth day after acceleration exposure. The water maze was a circular pool (50 cm in height, 150 cm in diameter) with painted black inner wall, which was filled with 25 cm high water and kept at 22–25°C. The pool was divided into four quadrants (quadrants I–IV) according to four equal distance points on inner wall. An escape platform painted black (23 cm in height, 12 cm in diameter) was submerged 2.0 cm under the water surface and placed at the center of quadrant I in the pool. Three different starting points for rats were placed around the perimeter of the pool. The swimming traces of rats were recorded by a camera suspended over the center of the pool and were transmitted to information collection and conduction system manufactured by Medical Science Academy of China on a computer. The experimenter always sat at the same position during the tests.

#### 2.4.1. Escape Acquisition

At the beginning of the task, the rats were placed on the platform and permitted to remain on it for 15 s to be trained to remember the platform. After that, they were placed into the water against the pool wall at one of three starting points of three quadrants except the platform quadrant. The order of the start position varied in every trial. From the eighth to fourteenth days, each rat performed three consecutive training trials, with an intertrial interval of 60 s, per day. Rats were allowed to search for the platform during a period of 180 s. In the case of failing to escape, they were gently guided to the platform and remained there for 15 s and their escape latency was recorded as 180 s. The escape latency was recorded as a measure for spatial memory in the escape acquisition.

#### 2.4.2. Probe Trial

On the day after the last training trial in the escape acquisition test, the rats were submitted to the probe trial in which the platform was removed. In the 180 s probe trial, swimming patterns were analyzed with respect to a target annulus consisting of a 20 cm diameter circular area centered at the previous target location. The swim path of each rat was recorded, and the percentage of time spent in the target annulus and the number of times the target annulus was crossed during the probe were recorded for each rat.

### 2.5. Preparation of Tissue Samples

Following the last MWM test, rats were anaesthetized with pentobarbital-sodium and the whole brains were removed quickly after decapitation. The hippocampus was dissected carefully and placed in the precooled homogenate medium by weight/volume ratio of 1 : 9 to make 10% tissue homogenate and then centrifuged at 3500 rpm/min at 4°C for 10 min. Protein concentration of the supernatant was determined by incubation in bicinchoninic acid protein assay reagent (BCA) containing 0.1% Triton X-100 for 30 min at 37°C. The reaction was stopped by adding 1 M NaOH, and the absorbance was measured at 550 nm [20].

### 2.6. Determination of the Activities of AChE, ChAT, SOD, and GSH-Px and the Content of ACh and MDA

The activities of enzymes SOD, GSH-Px, AChE, and ChAT and the levels of ACh and MDA in the hippocampus homogenate were quantified by using the corresponding detection kits according to the manufacturer's instructions. Nitrite method was used to determine SOD activity with a wavelength of 550 nm to determine absorbance [21]. GSH-Px activity was measured at 412 nm by quantifying the rate of oxidation of reduced GSH to oxidized glutathione [22]. Thiobarbituric acid colouration was used to determine MDA concentration with wavelength 532 nm to determine absorbance [23]. AChE activity was measured as described by Kang et al. [24]. ChAT activity was determined according to the spectrometric method [25]. ACh level was measured using the method of Hestrin [26].

### 2.7. Western Blot Analysis

Protein extract was applied to western blot analysis. For this purpose, the hippocampus was homogenized in lysis buffer containing complete protease inhibitor cocktail (1 M Tris-HCl (pH 8.0), 5 M NaCl, 10% Nonidet P-40, and 1 M 1,4-dithio-DL-threitol (DTT)) [27]. Lysate samples containing 30 *μ*g of protein were fractionated by SDS-10% polyacrylamide gel electrophoresis and then blotted onto polyvinylidene fluoride membranes (Millipore, USA) with a transfer unit (Bio-Rad, USA). For the quantification of AChM1 receptor protein, these polyvinylidene fluoride membranes were thereafter incubated with primary antibodies as rabbit anti-rat-M1 polyclonal antibody at a 1 : 2000 dilution (Abcam, UK) and then incubated with a horseradish peroxidase-conjugated secondary antibody at a 1 : 5000 dilution. The membranes were put into chemiluminescence (ECL) reagent and exposed to film. Band densities were determined with image densitometer software. GAPDH was used as a housekeeping protein to normalize the protein load.

### 2.8. RNA Extraction and Quantitative Real-Time Polymerase Chain Reaction (qRT-PCR)

Total RNA in hippocampus tissue was isolated using TRIzol reagents (Invitrogen, USA). DNase I was used to remove genomic DNA residual from RNA samples. After that, 3 *μ*g of total RNA was converted into first-strand cDNA using the first-strand cDNA synthesis kit (Promega, USA) and oligo-d (T) 18 primers following the protocol recommended by the manufacturer. The real-time PCR primers for target transcripts were designed using the complete cDNA sequences deposited in GenBank (Table 1). The quantitative real-time PCR was carried out using the ABI PRISM 7300 Sequence Detection System (Applied Biosystems, USA) and analyzed with GeneAmp 7300 SDS software. In brief, real-time PCR reactions were performed with a 10 *μ*L reaction solution containing 1 *μ*L first-strand cDNA, 5 *μ*L 2x SYBR Green Master (Rox, Germany) Mix, 0.5 *μ*L of each forward and reverse primer, 3 *μ*L DNase, and RNase-free H_2_O. The thermal cycling conditions included 2 min at 50°C and 10 min at 95°C, followed by 40 cycles at 95°C for 15 s and 60°C for 1 min. The data were analyzed using ABI 7300 software. The AChM1 receptor and GAPDH transcript levels were estimated by using the formula 2^−ΔΔCT^ where ΔCT represents the difference in CT values between target gene and GAPDH.

#### 2.8.1. Data Analysis

All results are expressed as mean ± standard deviation (SD). Comparisons among groups were made using one-way analysis of variance (ANOVA) followed by least significant difference (LSD) post hoc multiple comparisons test using the statistical software package SPSS 16.0 for Windows (SPSS Inc., Chicago, IL, USA). Statistical differences of *P* < 0.05 were considered to be significant.

## 3. Results

### 3.1. EGb761 Attenuated the Memory Impairments Induced by Repeated High Sustained +Gz

**Figure 1:**
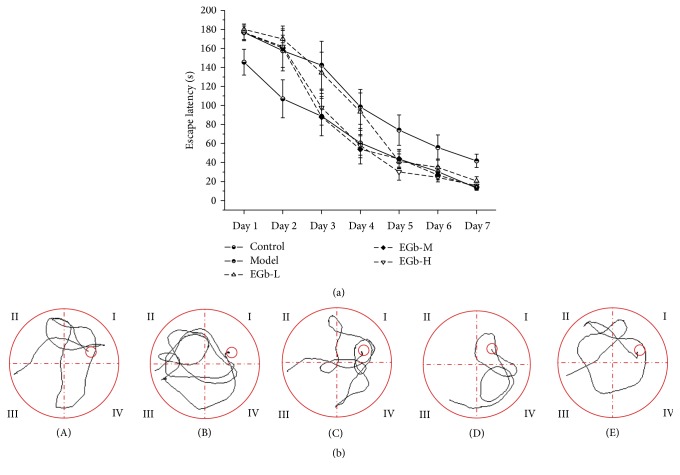
(a) Effects of EGb761 on escape latency of water maze test in rats. Data are shown as mean ± SD (*n* = 12 in each group). (b) Representative examples of search patterns in escape acquisition in the MWM test during a 180 s trial on day 7 in each group: (A) control; (B) model; (C) EGb-L; (D) EGb-M; (E) EGb-H.

The effect of EGb761 on spatial learning and memory was investigated through the MWM test. Firstly, we observed that the escape latency of all groups became shorter from day 1 to day 7, which is consistent with the fact that the learning and memory abilities of rats for the location of platform were gradually consolidated (Figure 1(a)). However, compared with the control group, the escape latency of model group was significantly longer in all test points (*P* < 0.05), which indicated that repeated high sustained +Gz exposure damaged the ability of learning and memory of rats. Secondly, EGb761-treated groups have decreased escape latency, especially for the EGb761-M and EGb761-H groups. From day 3, the escape latency of these two groups was significantly shorter than that of model group (*P* < 0.05). For EGb761-L group, a similar trend was observed from day 5. Interestingly, there was no difference between control group and EGb761-M/EGb761-H group on day 7 (*P* > 0.05).  Figure 1(b) shows the schematic trails of search patterns in the MWM on day 7 of escape acquisition. EGb761-treated rats displayed superior search patterns with shorter latencies and distances to find the platform, which was similar to the control group. Taken together, these results proved that EGb761 is able to protect the cognitive impairment caused by repeated high sustained +Gz.

**Figure 2:**
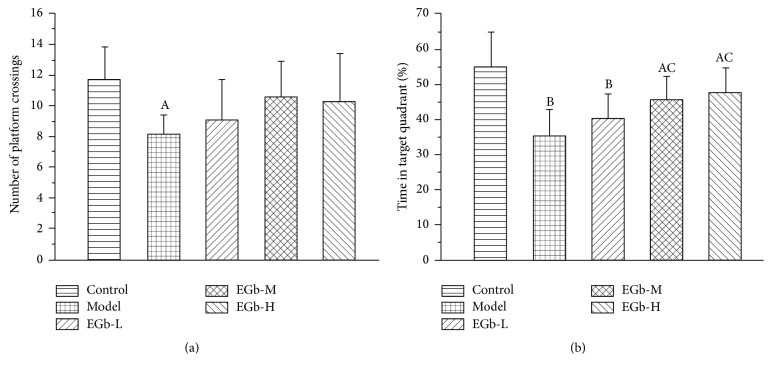
(a) Effects of EGb761 on the number of platform crossings of water maze test in rats. Data are shown as mean ± SD (*n* = 12 in each group). ^A^ *P* < 0.05 versus control group. (b) Effects of EGb761 on the percentage of time spent in the target quadrant during the probe trial test of water maze test in rats. Data are shown as mean ± SD (*n* = 12 in each group). ^A^ *P* < 0.05, ^B^ *P* < 0.01 versus control group; ^C^ *P* < 0.01 versus model group.

This was also verified by the percentage of time in the target annulus and the number of times crossed over the target annulus during the probe trial. In model group, both of these factors were significantly decreased compared with those of control group (Figures 2(a) and 2(b), *P* < 0.05). However, EGb761 administration can release the decrease of these two factors. EGb761 increased the number of platform crossings and time in target annulus in a dose-dependent manner and rats in EGb761-M and EGb761-H groups spent more time in the target annulus than those in control group (*P* < 0.01).

### 3.2. EGb761 Increased the Activity of ChAT and the Content of ACh but Reduced the Activity of AChE in Rats under Repeated High Sustained +Gz Exposure

**Figure 3:**
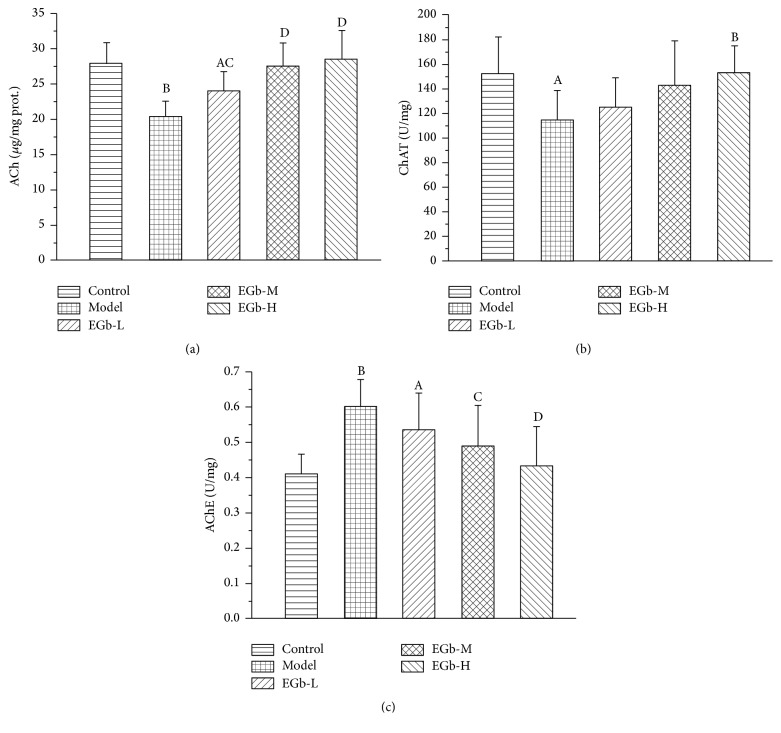
(a) Effects of EGb761 on the content of ACh in the hippocampus in rats. Data are shown as mean ± SD (*n* = 12 in each group). ^A^ *P* < 0.05, ^B^ *P* < 0.01 versus control group; ^C^ *P* < 0.05, ^D^ *P* < 0.01 versus model group. (b) Effects of EGb761 on the activity of ChAT in the hippocampus in rats. Data are shown as mean ± SD (*n* = 12 in each group). ^A^ *P* < 0.05 versus control group; ^B^ *P* < 0.01 versus model group. (c) Effects of EGb761 on the activity of AChE in the hippocampus in rats. Data are shown as mean ± SD (*n* = 12 in each group). ^A^ *P* < 0.05, ^B^ *P* < 0.01 versus control group; ^C^ *P* < 0.05, ^D^ *P* < 0.01 versus model group.

Given the important functions of cholinergic system in cognitive process, we investigated ACh content and AChE and ChAT activities in hippocampus of rats. As is shown in Figure 3(a), the content of ACh in model group was lower than that of control group (*P* < 0.01). However, EGb761-treated group exhibited higher content of ACh than that of the model group (*P* < 0.05, *P* < 0.01). For the enzymes of decomposition and synthesis of ACh, AChE activity increased, but ChAT activity decreased in the hippocampus under repeated high sustained +Gz exposure. In addition, EGb761 can significantly reverse these enzyme activities (*P* < 0.05, *P* < 0.01; Figures 3(b) and 3(c)).

### 3.3. EGb761 Upregulated the Expression Levels of Protein and mRNA of AChM1 Receptor in Rats under Repeated High Sustained +Gz Exposure

**Figure 4:**
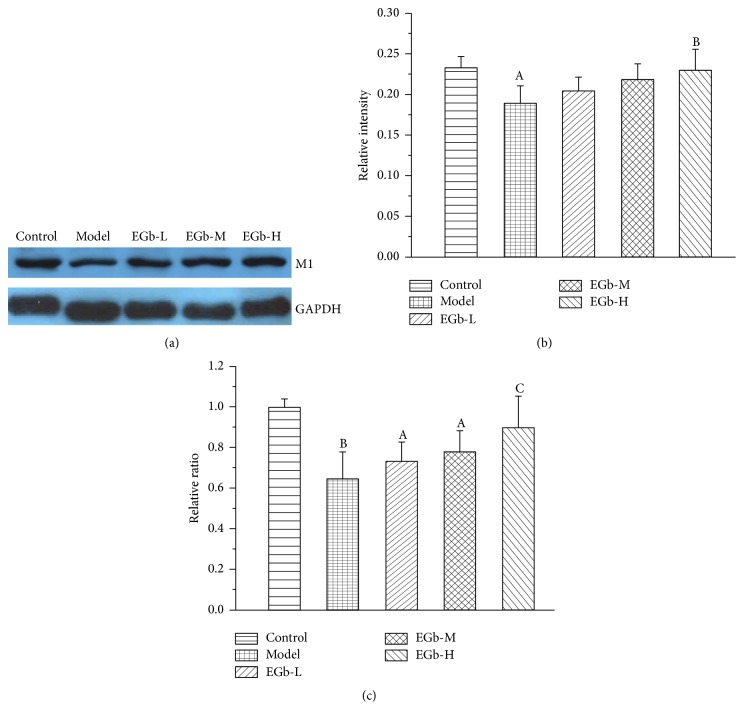
(a) The expression of AChM1 receptor protein in hippocampus. Representative polyacrylamide gel electrophoresis photograph from each group; (b) western blot analysis of the effect of EGb761 on the expression of AChM1 receptor protein in the hippocampus in rats. Data are shown as mean ± SD (*n* = 5 in each group). ^A^ *P* < 0.05 versus control group; ^B^ *P* < 0.05 versus model group. (c) qRT-PCR analysis of the effect of EGb761 on the expression of AChM1 receptor mRNA in the hippocampus in rats. Data are shown as mean ± SD (*n* = 5). ^A^ *P* < 0.05, ^B^ *P* < 0.01 versus control group; ^C^ *P* < 0.05 versus model group.

In addition to the ACh content, the protein and mRNA levels of AChM1 receptor were also evaluated. As is shown in Figures 4(a)–4(c), compared with control group, the AChM1 receptor protein level in the model hippocampus was significantly decreased. EGb761 upregulated AChM1 receptor expression in a dose-dependent manner and its level in EGb761-H group was significantly higher than that in model group. In addition, the variation trend of AChM1 receptor mRNA was consistent with its protein level.

### 3.4. EGb761 Increased the Activities of SOD and GSH-Px but Reduced the Content of MDA in Rats under Repeated High Sustained +Gz Exposure

**Figure 5:**
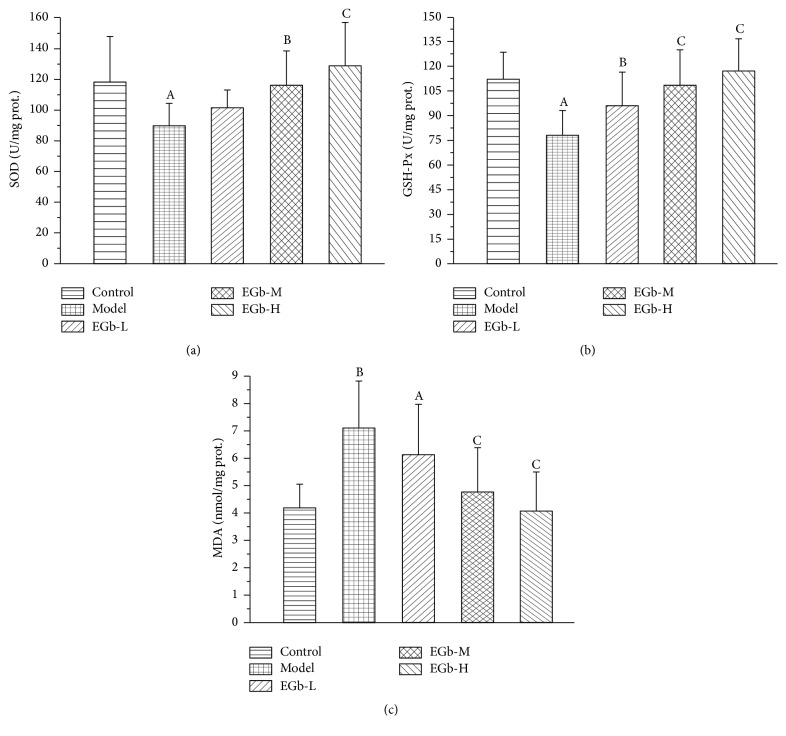
(a) Effects of EGb761 on the SOD activity in the hippocampus in rats. Data are shown as mean ± SD (*n* = 12 in each group). ^A^ *P* < 0.05 versus control group; ^B^ *P* < 0.05, ^C^ *P* < 0.01 versus model group. (b) Effects of EGb761 on the GSH-Px activity in the hippocampus in rats. Data are shown as mean ± SD (*n* = 12 in each group). ^A^ *P* < 0.01 versus control group; ^B^ *P* < 0.05, ^C^ *P* < 0.01 versus model group. (c) Effects of EGb761 on the MDA level in the hippocampus in rats. Data are shown as mean ± SD (*n* = 12 in each group). ^A^ *P* < 0.05, ^B^ *P* < 0.01 versus control group; ^C^ *P* < 0.01 versus model group.

The oxidative stress and antioxidant status were analyzed to determine the role of oxidative damage induced by repeated high sustained +Gz exposure. From the results (Figures 5(a)–5(c)), we observed that the activities of SOD and GSH-Px in hippocampus of the rats in the model group were significantly decreased, while the MDA content was significantly increased compared with the control group (*P* < 0.05). Treatment with EGb761 restored the SOD and GSH-Px activities in a dose-dependent manner, and in EGb761-M and EGb761-H groups both of them were much higher than those in control group (*P* < 0.05, *P* < 0.01). Accordingly, the concentrations of MDA in EGb761-treated groups were decreased in different degree.

## 4. Discussion

In this study, we observed that repeated high sustained +Gz exposure impaired the learning and memory ability of rats, which can be described as increased escape latency, and decreased the numbers of crossing platform areas and the time spent in the target quadrant in MWM test. This impairment of cognition induced by repeated high sustained +Gz exposure is supposedly similar to the damage of ischemia-reperfusion owing to the fact that the brain may produce ischemia under +Gz condition. Ischemia-reperfusion causes reactive oxygen species overproduction and cholinergic system dysfunction [28–30]. Cholinergic signaling pathway is involved in central cognitive processes such as learning and memory [31–33]; cholinergic deficit is a major neuropathological feature that is associated with memory loss and closely correlated with the severity of cognitive dysfunction in AD [34] and poststroke cognitive impairments [35, 36]. Oxidative stress can alter brain activity including neurotransmission, cause neuronal cell death, and reduce the ability of learning and memory [37–39]. Many antioxidants have a protective effect against learning and memory deficits [40]. In this study, we examined the antioxidant and cholinergic systems in the hippocampus, since it is essential for the regulation of spatial learning and memory processes in animals [41]. Therefore, the alternation of cholinergic system and oxidative stress in this region may contribute to the impairment of learning and memory.

As a key transmitter in cholinergic system, ACh plays an important role in the process of learning and memory [42]. There is a positive correlation in rat hippocampus between increased levels of ACh and improved spatial memory performance in a maze task [43–45]. ChAT and AChE are two key enzymes that regulate the availability of ACh [46]. ChAT involves the synthesis of acetylcholine. The degree of cognitive dysfunction in AD patients is significantly correlated with decline in ChAT activity and loss of cholinergic neurons, while AChE acts to degrade ACh to acetate and choline in the synaptic cleft and eventually terminates cholinergic transmission [47]. We found that repeated high sustained +Gz exposure resulted in a significantly increased activity of AChE but decreased activity of ChAT, as well as a significant decrease in the content of ACh in hippocampus of rats, which indicated that repeated high sustained +Gz exposure causes the central cholinergic system dysfunction.

In addition, we were also interested in the muscarinic acetylcholine receptors, which control the time course of evoked ACh release and play an essential role in memory formation [48]. So far, at least five subtypes of muscarinic acetylcholine receptor (M1–M5) have been found in the brain and were well characterized, in which M1 and M2 are mainly expressed in the hippocampus and cortex [49, 50]. The M1 receptor is viewed as the most important subtype for memory and attention mechanisms [51, 52]. We investigated the effect of repeated high sustained +Gz exposure on the status of the mRNA and protein expression levels of AChM1 receptor. The results showed that repeated high sustained +Gz exposure caused lower expression level of AChM1 receptor both on the protein and on mRNA. All these implied that repeated high sustained +Gz exposure affected cholinergic system extensively.

The increase in reactive oxygen species (ROS) production is one of the key events in I/R injury [53], which may be also applicable to repeated high sustained +Gz exposure. During tissue ischemia, a reduction in the availability of ATP results in the degradation on adenosine diphosphate (ADP), adenosine monophosphate [54], adenosine, inosine, and hypoxanthine. Furthermore, xanthine dehydrogenase is converted to xanthine oxidase. Xanthine oxidase relies on oxygen to metabolize hypoxanthine, and when this is provided by reperfusion (reoxygenation), ROS molecules are formed, with a large capacity to cause injury to tissue [55]. SOD and GSH-Px are the two main enzymes involved in cellular protection against damage caused by oxygen-derived free radicals and are also the classical indexes to evaluate the antioxidative effects [56]. MDA, the degradation product of the oxygen-derived free radicals and lipid oxidation, reflects the damage caused by reactive oxygen species [57]. In this study, the decreased activities of SOD and GSH-Px and the increased content of MDA in the hippocampus of rats from model group implied that hippocampus neurons were subjected to oxidative stress, which may induce degeneration of neurons and lead to learning and memory deficits.

EGb is widely viewed as a memory-enhancing agent. High doses of EGb (200 mg/kg) scavenge free radicals [58], medium doses (100 mg/kg) promote short-term memory in passive avoidance tests in aged mice [59], and lower doses (50 mg/kg) promote spatial learning in aged rats [60]. In the present study, we observed the effects of EGb761 at three doses on impairment of learning and memory induced by high sustained +Gz. In MWM test, all three dosages of EGb decreased escape latency and increased the numbers of crossing platform areas and the time spent in the target quadrant, but effects of high doses and medium doses were observed on the third day and those of the low doses were observed on the fifth day. On the seventh day, three dosages had no significantly different effects. The results suggested that EGb761 has a protective effect on impairment of learning and memory induced by repeated high sustained +Gz in a dose-dependent manner.

It was documented that EGb enhances the ability of learning and memory by increasing ACh level and inhibiting AChE activity [61, 62]. In this study, we found that EGb761 can regulate the activity of AChE, as well as ChAT, and increased the content of ACh. Compared with control group, the content of ACh and the activity of ChAT in EGb-H group were significantly higher, while the activity of AChE became lower. Therefore, the role of EGb761 in the activity of ChAT and AChE and the level of ACh may be involved in the improvement of learning and memory in rats exposed to repeated high sustained +Gz. Moreover, EGb also could upregulate the AChM1 receptor mRNA and protein expression in a dose-dependent manner, and the level in EGb-H groups was significantly higher than that in model group. Thus, it can be deduced that EGb-induced cognitive ameliorative effects are likely related to controlling ACh release via regulating the expression of muscarinic ACh receptors. Previous reports suggested that* Ginkgo biloba* may have a particular effect on cholinergic receptors and enhance effective cognitive ability. Administration of scopolamine, a muscarinic antagonist, can transiently blockade the cholinergic muscarinic receptors and cause impairments in memory and other aspects of cognitive performance, but this impairment induced by scopolamine is reduced by* Ginkgo biloba* [15]. Taylor verified that chronic administration of* Ginkgo biloba* can increase cholinergic muscarinic receptors [63]. Taken together, we believed that EGb functions with multiple action modes in the cholinergic pathway.

EGb761 contains two major groups of active substances, flavone glycosides and terpene lactones [64]. These constituents have the ability to scavenge free radicals and the antioxidant properties are considered to contribute to the neuroprotective potential of EGb761 against neuronal injury [65, 66]. In the present study, EGb significantly ameliorated abnormalities of SOD and GSH-Px activities and reversed the content of MDA. The results indicate that EGb is likely to increase the oxidation resisting capacity of the hippocampus, subsequently preventing the hippocampus neurons from lipid peroxidation damage.

Besides the efficacy on cholinergic system and the antioxidant action,* Ginkgo biloba*'s neuroprotective and cognitive-enhancing effects were also related to its platelet-activating factor (PAF) antagonistic effects. The ginkgolides have been shown to be antagonists of PAF, which has proinflammatory effects such as increasing vascular permeability [67] and direct effects on neuronal function and long-term potentiation [68, 69]. It has also been shown that EGb761 affects a number of neurotransmitter systems except for cholinergic system, including serotonergic, adrenergic, dopaminergic, GABAergic, and glutamatergic systems [70–72]. In addition, during high sustained +Gz exposure, a deficiency in cerebral blood transport leads to a decrease in oxygen and nutrient supply to the brain. Several groups have demonstrated that EGb761 treatment can increase cerebral blood flow and alter cell energy metabolism following the induction of hypoxia/ischemia [73–76], which may also help to protect the ability of learning and memory from impairment. Apart from these considerations, the effects on cognition-improving action and precise mechanism of EGb761 in rats exposed to repeated high sustained +Gz remain unclear and are an interesting topic to be further explored.

In summary, in this study, firstly, we showed that repeated high sustained +Gz can cause learning and memory impairment in rats. We further evaluated the protection efficacy of EGb against these learning and memory damage instances and interpreted the multiple molecular actions that involve the protection. In detail, this includes the increasing ACh production by the promotion of ChAT, the inhibition of AChE, and the upregulation of AChM1 receptor expression together with antioxidant enzymes activities. EGb merits further exploration as a potential antigravitation agent to maintain the flying security of pilots.

## Figures and Tables

**Table 1:** Sequences of the primers for mRNA of AChM1 receptor and GAPDH by real-time PCR.

Genes	Primer sequence	Product size (bp)	Accession number
AChM1	AGTTCCTCTCCCAACCCATC	161	NM_080773.1
	ACCTTTGCCTGGTGTCTCAG		

GAPDH	GGTCGGTGTGAACGGATTTGG	148	BC059110
	GCCGTGGGTAGAGTCATACTGGAAC		
